# Mindfulness-based cognitive therapy for multiple chemical sensitivity: a study protocol for a randomized controlled trial

**DOI:** 10.1186/1745-6215-13-179

**Published:** 2012-09-27

**Authors:** Christian Riise Hauge, Peter Jens E Bonde, Alice Rasmussen, Sine Skovbjerg

**Affiliations:** 1 The Danish Research Centre for Chemical Sensitivities, Department of Dermato-Allergology, Copenhagen University Hospital Gentofte, Ledreborg Alle 40, 2.th, Gentofte, 2820, Copenhagen, Denmark; 2 Department of Occupational and Environmental Medicine, Copenhagen University Hospital Bispebjerg, Copenhagen, Denmark; 3 Psychiatric Centre, Copenhagen University Hospital Bispebjerg, Copenhagen, Denmark

**Keywords:** Mindfulness-based cognitive therapy, Multiple chemical sensitivity, Randomized controlled trial

## Abstract

**Background:**

Multiple chemical sensitivity (MCS) is a condition characterized by recurrent, self-reported symptoms from multiple organ systems, attributable to exposure to a wide range of chemically unrelated substances at low levels. The pathophysiology is unknown, and affected individuals generally favor avoidance of the symptom triggering substances as a coping strategy. The impact of MCS on daily life may thus be severe. An intervention that may effectively reduce the impact of MCS, alleviate the symptoms and the psychological distress associated with the condition is therefore highly needed. In this study we will assess the effects of a mindfulness-based cognitive (MBCT) program on MCS.

**Methods/Design:**

Using a randomized controlled design (RCT), we will compare MBCT with treatment as usual (TAU). The MBCT intervention will include 8 weekly 2.5 hour sessions, and 45 minutes of mindfulness home practice 6 days each week. Participants will be asked to complete questionnaires at baseline, post-treatment, and at 6 and 12 months’ follow-up. Based on sample size estimation, 82 participants will be randomized to either the MBCT intervention or to TAU. The primary outcome will be a measure of the impact of MCS on the participants’ lives. The secondary outcome measures are physical symptoms of psychological distress, perceived stress, illness perceptions, QOL, and work ability. Lastly, we will assess whether any effect of MBCT on the primary effect measure is mediated by level of mindfulness, self-compassion, perceived stress, and rumination.

**Discussion:**

This trial will provide important information on the effects of MBCT on MCS.

**Trials registration:**

Clinical trials identifier NCT01240395

## Background

The term ‘multiple chemical sensitivity’ (MCS) is applied to a condition of recurrent, self-reported symptoms from multiple organ systems. Other labels have been ascribed to the symptoms, but MCS is widely used in the scientific literature, and thus, will be used here without reference to any assumptions about etiology. The symptoms follow perceived exposure to a wide range of airborne chemicals at levels normally considered non-toxic [1]. Frequently reported symptom-eliciting chemicals and environmental agents include fragranted products, motor-vehicle exhaust fumes, cleaning agents, freshly printed papers or magazines, and smoke from wood burners [2,3]. A recent population-based study showed that extreme fatigue, headache, gastrointestinal symptoms, muscle and joint pain, upper airway symptoms, and irritability are the most frequently reported symptoms [3]. It has been documented that symptoms from the central nervous system (CNS) other than headache are the single most predictive factor of the severity of MCS [2]. Despite the limited number of studies investigating syndrome stability in MCS, evidence points to MCS being a chronic condition [4,5].

The pathophysiological mechanisms behind MCS remain unknown [6,7]; however, current evidence suggests that MCS is more likely to be due to individual susceptibility factors than to a toxicological response to common airborne chemicals [7,8]. Moreover, recent evidence indicates that sustained levels of arousal may be a risk factor in the development of MCS [9]. Several underlying mechanisms have been proposed, such as sensitization of the CNS [10,11], and symptom acquirement due to classical conditioning [12,13]. Increased levels of depression, anxiety, and somatization, are frequently reported [14-17], and it has thus been suggested that MCS belongs to a spectrum of somatoform disorders [18]. However, evidence supporting this view is sparse.

Provocation studies examining responses of individuals with MCS when exposed to either active or sham provocation have found that, in general, these individuals cannot differentiate between active and sham exposures, symptomatically or otherwise. While the mechanisms by which airborne chemicals trigger MCS symptoms are unclarified, the significance of mental representations of illness has been found to be particularly influential in patients presenting with medically unexplained symptoms, in terms of onset, persistence of symptoms, and degree of disability [19], when comparing a medically unexplained illness with an illness of known physiological origin [20].

Currently, there are no evidence-based treatments or clinical guidelines for MCS [21,22]. A typical coping response seems to be that of avoiding potential chemical triggers, typically through the creation of a safe living space and the application of protective routines, thus avoiding exposure to symptom-eliciting stimuli [23,24]. However, this may come at a cost, because several aspects of the patients’ everyday life, including lifestyle, social relations, and occupational conditions, are affected by the behavior [23]. Considering the impact MCS has on the lives of those affected, an effective evidence-based treatment is urgently needed.

Mindfulness, defined as ‘the awareness that emerges through paying attention on purpose, in the present moment, and non-judgmentally to the unfolding of experience moment by moment’ [25], is becoming increasingly popular, and mindfulness-based interventions show promise in the treatment of stress-related medical disorders [26]. It has been suggested that mindfulness-based interventions could be useful in the treatment of such disorders by improving disease management and reducing psychological distress, thereby improving wellbeing [27].

A few clinical trials have assessed the effect of mindfulness-based therapy on conditions with hypothesized shared illness mechanisms and a high degree of co-morbidity to MCS; such as fibromyalgia [28,29], irritable bowel syndrome [30] and chronic fatigue [31]. Although the results of these studies are inconclusive, the findings are generally positive, warranting larger randomized clinical trials (RCTs).

One study, applying a non-randomized waiting-list design, evaluated the effect of a mindfulness-based intervention program on participants with MCS with various types of co-morbidity to other functional disorders [32]. The results showed that the mindfulness-based intervention was associated with a significant decrease in symptoms of somatization and psychological distress compared with the waiting-list control group. However, the study did not include outcome measures directly related to the participants’ MCS, so it is uncertain whether the intervention had any effect on this outcome. The feasibility of a MBCT program for MCS was tested in a recent randomized pilot trial. The study concluded that a group intervention was feasible for MCS and a larger randomized trial could be considered [33].

### Objectives

The main aim of the proposed study is to evaluate whether an 8-week mindfulness-based intervention program is associated with a reduction in the degree to which MCS affects participants’ lives (for example, ability to travel, to be around others and to enjoy social activities). The secondary aims are to evaluate whether the intervention is associated with a reduction in commonly experienced symptoms (for example, muscle or joint aches, headache, difficulty in concentrating), severity of reactions to common chemicals, psychological distress (anxiety, depression, and somatization), and perceived stress, and whether the intervention increases participants’ quality of life (QOL) and work ability. The study will also assess whether the intervention affects the participants’ illness perceptions. Finally, it will assess whether degree of mindfulness, stress, self-compassion, and rumination are possible mediators of a treatment effect.

#### Hypotheses

The primary hypothesis is that the degree to which MCS affect participants’ daily lives will be significantly reduced in the mindfulness intervention group compared with the control group. The secondary hypotheses are 1) that we expect a significant alleviation of MCS symptoms, symptoms of depression, anxiety, somatization, and perceived level of stress, thereby improving overall psychological wellbeing compared with that in the control group, and 2) that the mindfulness intervention will lead to positive changes in illness perception and will improve QOL and work ability.

In terms of mediating treatment mechanisms, we expect that a treatment effect will be mediated by the level of mindfulness, as was found in a previously conducted trial with participants with a similar level of disability [30]. Additionally, we will assess whether self-compassion, which was recently found to mediate a reduction in depressive relapse [34], also mediates a treatment effect in our group of patients. Moreover, and in line with a previous prospective study that established prolonged stress as a risk factor in the development of symptoms [9], we will investigate whether the reverse is also true; that is, whether a reduction in the level of stress is associated with a reduction in the degree to which chemicals affect participants’ daily lives. Furthermore, we will explore whether a positive change in illness perceptions (for example, a greater sense of control, an increased sense of understanding of MCS, and a reduction in the concern associated with MCS) will mediate a treatment effect. Lastly, recent research suggests that rumination could be a transdiagnostic factor underlying several psychiatric conditions [35], and we will investigate whether it is also a possible mediator of a treatment effect for individuals with MCS.

## Methods/Design

### Empirical design

The study is an RCT in which the effect of a mindfulness-based intervention program will be compared with that of a TAU control group. Because this is a pragmatic trial, we will enhance the external validity by keeping exclusion criteria to a minimum. The study will be undertaken in the cities of Copenhagen and Aarhus, Denmark.

### Participants

Participants will be recruited through several sources. First, we will contact GPs in the Copenhagen and Aarhus areas and provide them with information about the study. Second, we will contact individuals with MCS registered at the Danish Research Centre for Chemical Sensitivities who have agreed to be contacted about research projects, and invite them to participate in the study. Lastly, we will advertise the study on the website of the Danish Research Centre for Chemical Sensitivities, in patient magazines, and in local newspapers.

### Inclusion criteria

Participants will need to be aged 18 to 65 years, and provide written, informed consent. They will need to fulfill the expanded consensus criteria for MCS [36,37], as follows. 1) The condition has lasted for at least 6 months causing significant lifestyle or functional impairments; 2) there are reproducible CNS symptoms and 3) at least one symptom from another organ system; 4) the symptoms occur in response to low levels of exposure to 5) multiple unrelated chemicals and 6) improve or are resolved when these inciting substances are removed.

### Exclusion criteria

The exclusion criteria are: presence of 1) a psychotic or bipolar disorder, 2) suicidal ideations, 3) drug or alcohol abuse; and 4) previous engagement in a mindfulness-based intervention program.

### Assessment

Before randomization, all participants will be assessed, using the Schedules for Clinical assessment in Neuropsychiatry (SCAN), an instrument to assess and classify the psychopathology and behavior associated with major psychiatric disorders. The interview contains an extensive section on somatic symptoms, providing an opportunity to obtain an overview of the participants’ symptoms from various organ systems. We will also use SCAN to assess whether the participants fulfill the criteria for various other functional somatic syndromes, applying the SCAN algorithms suggested by Fink and Schröder [38]. Classification of such syndromes will be based solely on the interview rather than by a thorough medical investigation, as the purpose is purely descriptive. Lastly, the SCAN interview will be used to identify subjects who present with severe psychopathology, and who thus will be excluded from participation.

Concerning the specific needs that chemically intolerant people may have in terms of environment, eligible participants will be asked to consider whether they are able to engage in a group setting and to refrain from using fragranced products while attending the classes.

### Randomization

The randomization will be carried out as follows. Once we have recruited 16 to 20 eligible participants (the number of participants required to start off a MBCT group) who have signed a written consent form, they will be randomized by a computer-generated allocation sequence either to intervention with MBCT or to the TAU control condition. The randomization will be pre-programmed so as to produce equal numbers in both groups. The randomization procedure will be carried out at the Research Centre for Chemical Sensitivities by a researcher who is otherwise not involved in the trial, thus securing concealment to the research team. Once the randomization has been carried out, the first author will contact participants by telephone and email to inform them about the allocation.

### Intervention

The mindfulness-based intervention in this RCT is modeled on MBCT, which is a group skills-based training approach developed to prevent relapse of depressive episodes [39]. MBCT is partly based on the mindfulness-based stress reduction (MBSR) program developed by Kabat-Zinn [40], and partly on cognitive therapy for depression. MBCT focuses on the aspect of ‘de-centering’, meaning not accepting the content of thoughts as facts and not identifying with thoughts [39]. The MBCT program has been fully manualised by Segal and colleagues, describing the exercises and rationale in detail [41]. However, as the original program was developed to prevent relapse of depression, some minor modifications will be made to the curriculum in this trial to adapt it for individuals with MCS. For example, session seven in the MBCT program deals with identifying signs of depressive relapse, but in our study, the emphasis will be on applying mindfulness in coping with stress. The main treatment component will be mindfulness exercises, which includes various forms of meditation and yoga. The purpose of the mindfulness exercises is to cultivate the ability to stay in the present with full awareness, and to practice an attitude of acceptance of whatever sensation, pleasant or unpleasant, that arises in the present moment. Before commencing the program, all the participants will be invited to an individual session with the group therapist with the aim of getting to know the participants and answering questions about the program. The program will be given to groups with a maximum of 15 participants, and will comprise eight weekly sessions, each 2.5 hours long, plus a half-day of silent retreat between weeks 6 and 8. Additionally, during the program, participants will be encouraged to do home work assignments of up to 45 minutes 6 days a week. Guided instructions will be provided on a CD for home practice. If a participant is unable to attend a session, they will be e-mailed any written material handed out during the class. Finally, participants will be offered follow-up sessions at months 1, 3, and 6 post-treatment.

The therapists teaching mindfulness in the trial will be two clinical psychologists, both of whom have extensive experience in delivering mindfulness-based treatment. They have undergone formal education in mindfulness-based therapies from The Oxford Mindfulness Centre and from the Umass Centre for Mindfulness in Medicine, Healthcare, and Society, respectively.

### Treatment as usual

The control condition in this trial is a TAU control group for comparison with the intervention group. TAU in this trial does not refer to a specific treatment regimen but rather to unconstrained services that may vary across participants. Participants randomized to the TAU group will be informed that they should continue receiving their usual care from their GP, specialist physician or other health professional(s) according to their needs. Control participants will be requested not to engage in any mindfulness program until the trial has been completed; nevertheless, as part of the post-intervention questionnaire, control participants will be asked whether they have initiated a mindfulness program themselves, providing an opportunity to control for confounding in the subsequent analyses should some have in fact done so. The treatments that the participants will receive will be registered for both the intervention group and control group as part of the trial.

### Measurements

All outcomes will be measured at baseline, post-treatment, and at the follow-ups at 6 and 12 months.

#### Primary outcome

##### The Quick Environmental Exposure and Sensitivity Inventory

The Quick Environmental Exposure and Sensitivity Inventory (QEESI) has been developed as a screening instrument for MCS designed to facilitate history-taking from individuals who report chemical intolerance [42]. It consists of five scales, of which this study will use the three: symptom severity, chemical intolerances, and life impact, each containing 10 items and producing a score ranging between 0 and 100. The Life Impact Scale (LIS) of the QEESI, which is the primary outcome measure, asks the participant to consider how much their reactions have affected various aspects of their life in terms of parameters such as diet, ability to go to work or school, ability to be around others and enjoy social activities, and relationship with spouse and family.

A Danish translation of the QEESI has been evaluated in terms of internal consistency, test-retest reliability, sensitivity, and specificity, and in order to establish normative data [43]. The psychometric properties of QEESI were found to be satisfactory, which is in accordance with other similar studies [42,44].

#### Secondary outcomes

##### Symptom-Check List-92

The Symptom Checklist (SCL-92) is a self-administered questionnaire for measuring psychological distress or the degree of affective distress. The questionnaire covers nine dimensions; however, only the somatization, depression, and anxiety scales will be used in this study. The items are measured on a five-point Likert scale. The SCL-92 has been translated into Danish, and has been psychometrically evaluated in a Danish population, showing that the non-psychotic scales function well and seem to reflect a single broad dimension of stress [45].

##### World Health Organization Quality Of Life, Brief Version

The World Health Organization Quality of Life (WHOQOL-BREF) is a relatively short multidimensional questionnaire, which is a generic measure of health-related quality of life. The questionnaire comprises the domains of physical health, psychological wellbeing, and social conditions and environment, as well as a general QOL domain. The questionnaire has been shown to have good psychometric properties and to adequately assess domains relevant to QOL in a large number of cultures worldwide [46].

##### Perceived Stress Scale

The Perceived Stress Scale (PSS) is designed to measure the degree to which situations in a person’s life are appraised as stressful [47]. The PSS was designed for use with community samples with at least a secondary school education. The items are easy to understand and the response options are simple to grasp [48]. The PSS has been adapted to a short version consisting of 10 questions, the PSS-10, which has proven to be a valid and reliable measure of perceived stress [48].

##### Brief Illness Perception Questionnaire

The Brief Illness Perception Questionnaire (Brief IPQ) has nine items and is designed to measure patients’ cognitive and emotional representations of their illness. Five items assess cognitive illness representations, two items assess emotional representations, and one item assesses illness comprehensibility. The items are rated using a response scale of 0 to 10. Each causal item is grouped into categories (for example, stress, lifestyle, hereditary factors), which are determined by the particular item studied. The Brief IPQ has been found to have good test-retest reliability and validity [49].

##### Role Work Functioning Index

The Role Work Functioning Index (RWFI) is a measure of work ability, a concept that refers to the balance between the demands of a job and the individual’s capacity to master these demands. As opposed to most instruments that assess work ability, the RWFI does not ask respondents to consider their health when responding to questions about their work ability. The index consists of seven questions tapping into the following dimensions of work demands: tempo; workload; and physical, social, and cognitive job demands (K. Thielen, 2011, personal communication).

### Mediators

#### Rumination-reflection questionnaire

The rumination subscale of the Rumination-Reflection Questionnaire is a 12-item questionnaire measuring the tendency to recurrently think about threats, losses, or injustices to the self [50]. The scale has been found to tap into a type of self-attentiveness closely linked to the personality trait of neuroticism. The authors reported an internal consistency coefficient of 0.90 for the rumination subscale.

#### Self-Compassion Scale

The Self-Compassion Scale measures the ability to have a healthy stand towards oneself that does not involve evaluations of self-worth [51]. Research has shown that self-compassion is associated with wellbeing and greater psychological health. The questionnaire used in this trial is a short form consisting of 12 questions, which has been shown to have a high level of correlation with the original questionnaire [52].

#### Five Facet Mindfulness Questionnaire

The Five Facet Mindfulness Questionnaire (FFMQ) assesses five general facets of being mindful in daily life: observing, describing, acting with awareness, non-reactivity to inner experience, and non-judging of inner experience. Items are rated on a five-point Likert scale ranging from 1 (never or very rarely true) to 5 (very often or always true). Previous studies have provided good support for construct validity of the FFMQ; furthermore, four of the five facets (except for ‘acting with awareness’) have been found to be significantly correlated with meditation experience [53].

### Statistical analysis

Statistical analyses will be conducted using the latest version of SPSS. Group allocation (intervention or control) will be concealed for the statistician who will perform the statistical analyses. Descriptive statistics for the two groups will be generated. In the statistical analyses of a possible effect, the mixed model repeated measures method will be used to test the effect of the intervention and the effect of time. Level of significance will be set at *P* < 0.05. All effect measures will be inspected for normality, and transformed as appropriate. Missing values will be handled by the multiple imputation method. Data analysis will be performed based on the intention-to-treat principle, meaning that all participants will be included in the analysis irrespective of their compliance with the intervention protocol. Lastly, an additional per-protocol analysis will also be conducted.

### Sample size

The sample size estimation is based on the primary effect measure, the QEESI. Results from a recently conducted study evaluating a Danish translation of the QEESI, showed that the patient sample had a mean value of 61.5 with a standard deviation of 24.3 on the LIS [43]. In this study, a clinical effect will be set as a 25% reduction on the LIS of the QEESI, which we regard as a clinically meaningful reduction in the effect of MCS on participants’ daily lives. With a reduction of 25% on the LIS, the study will need to include 41 experimental subjects and 41 control subjects to be able to reject the null hypothesis, that is, that the population means of the experimental and control groups are equal with probability (power) of 0.8. The type I error probability associated with this test of this null hypothesis is 0.05.

### Ethical considerations

The trial has obtained approval from the regional ethics committee (registration number H-2010-122), and is registered with the Danish Data Protection Agency.

There is no documentation of any side effects or serious risks related to participation in an MBCT program. However, participants may experience unpleasant thoughts and emotional reactions during the program, which we expect to deal with during the group sessions. If this is insufficient, the study therapists will be available for individual counseling, or they may refer participants to relevant treatment elsewhere if considered appropriate. Any adverse events reported by the participants will be registered and reported in future publications.

## Discussion

MCS is a condition that may seriously affect many aspects of the lives of afflicted individuals, and markedly reducing their health-related QOL [54,55]. Hence, an intervention that contributes to decreasing the negative effects of MCS is urgently needed. This study protocol describes a trial that will test MBCT as an intervention for MCS, with the primary aim of reducing the negative effects on the patient’s life caused by MCS. We will compare a group receiving MBCT with a control group receiving TAU. The study participants may seek out or continue any treatment they see fit while enrolled in the study. Participant behavior in terms of seeking other treatment (for example, medical or psychological) will be registered as part of the trial.

This study has several strengths. It is the first RCT to assess the effect of a mindfulness-based intervention for MCS. The results will be important in clarifying whether a mindfulness-based approach is indeed an effective method in reducing the negative effects of MCS on patients’ lives. Furthermore, the study will examine other effect measures that are likely to be affected by the participants’ intolerance, such as psychological distress, work ability, and QOL, and will thus provide valuable information on the effects of the intervention on these parameters as well. Moreover, the study will assess several potential mediating mechanisms of a possible treatment effect, which will be important in understanding the processes that underlie MBCT and what aspects of the intervention may be of particular importance for MCS.

The study also has some limitations. First, owing to the nature of the trial and the fact that the study comprises a TAU control condition, it is not possible to blind either the study participants or the therapists. However, the statistician conducting the analyses will be blinded to group allocation. Second, although we will carefully assess whether the study participants fulfill predefined criteria for MCS, these criteria are still based on self-report and there are no clinical tests available to verify that the participants’ symptoms are indeed due to MCS. Finally, it will not possible to include the most severe MCS cases in this study due to the nature of their symptoms, which prevent them from leaving their homes for prolonged periods.

## Trial status

The trial is ongoing and participants are currently being recruited.

## Abbreviations

Brief IPQ: Brief Illness Perception Questionnaire; CNS: Central nervous system; FFMQ: Five Facet Mindfulness Questionnaire; MBCT: Mindfulness-based cognitive therapy; MCS: Multiple chemical sensitivity; PSS: Perceived stress scale; QEESI: Quick environmental exposure and sensitivity inventory; RCT: Randomized controlled trial; SCAN: Schedules for clinical assessment in neuropsychiatry; SCL-92: Symptom Checklist-92; TAU: Treatment as usual.

## Competing interests

The authors declare that they have no competing interests.

## Authors’ contributions

All authors contributed to the study design. CH drafted the manuscript, and JPB, AR, and SS reviewed and revised the manuscript. All authors read and approved the final version.
